# *N*-(4,6-Dimethyl­pyrimidin-2-yl)-4-(oxolan-2-ylamino)benzene­sulfonamide

**DOI:** 10.1107/S1600536809043347

**Published:** 2009-10-28

**Authors:** Hadi D. Arman, Trupta Kaulgud, Edward R. T. Tiekink, David J. Young

**Affiliations:** a Department of Chemistry, The University of Texas at San Antonio, One UTSA Circle, San Antonio, Texas 78249-0698, USA; b Department of Chemistry, University of Malaya, 50603 Kuala Lumpur, Malaysia; c School of Biomolecular and Physical Sciences, Nathan, Griffith University, Queensland 4111, Australia

## Abstract

The title compound, C_16_H_20_N_4_O_3_S, adopts an l-shaped conformation, as seen by the dihedral angle of 76.93 (7)° formed between the two aromatic rings. The most notable feature of the crystal packing is the formation of N—H⋯O and N—H⋯N hydrogen bonds that lead to supra­molecular chains orientated along the *b* axis.

## Related literature

For background to the co-crystallization of active pharmaceutical agents, see: Shan & Zaworotko (2008 ▶). For background to sulfa drugs, see: Caira (2007 ▶); Nishimori *et al.* (2009 ▶). For the synthesis, see: Fructos *et al.* (2006 ▶); Kemnitz *et al.* (1998 ▶). For related studies on co-crystal formation, see: Broker & Tiekink (2008 ▶); Broker *et al.* (2008 ▶).
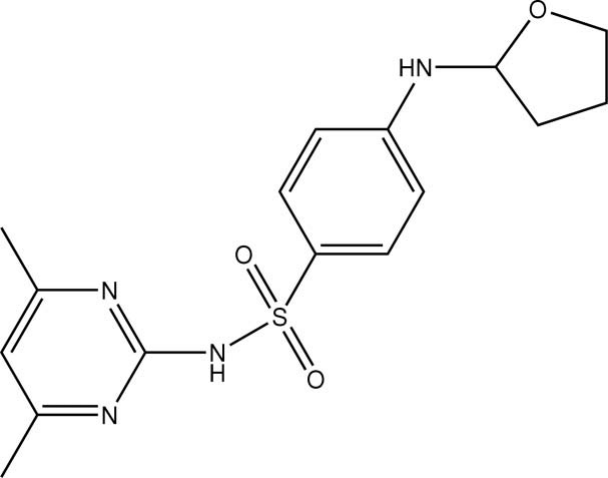


## Experimental

### 

#### Crystal data

C_16_H_20_N_4_O_3_S
*M* *_r_* = 348.42
Monoclinic, 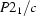

*a* = 10.291 (5) Å
*b* = 9.592 (4) Å
*c* = 17.196 (8) Å
β = 106.445 (10)°
*V* = 1628.0 (13) Å^3^
*Z* = 4
Mo *K*α radiation
μ = 0.22 mm^−1^
*T* = 98 K
0.35 × 0.21 × 0.11 mm

#### Data collection

Rigaku Saturn724 diffractometer
Absorption correction: multi-scan (*ABSCOR*; Higashi, 1995 ▶) *T*_min_ = 0.761, *T*_max_ = 1.000
11164 measured reflections
3749 independent reflections
3341 reflections with *I* > 2σ(*I*)
*R*_int_ = 0.046

#### Refinement

*R*[*F* ^2^ > 2σ(*F* ^2^)] = 0.057
*wR*(*F* ^2^) = 0.137
*S* = 1.10
3749 reflections
225 parameters
2 restraints
H-atom parameters constrained
Δρ_max_ = 0.60 e Å^−3^
Δρ_min_ = −0.39 e Å^−3^

### 

Data collection: *CrystalClear* (Rigaku/MSC, 2005 ▶); cell refinement: *CrystalClear*; data reduction: *CrystalClear*; program(s) used to solve structure: *SHELXS97* (Sheldrick, 2008 ▶); program(s) used to refine structure: *SHELXL97* (Sheldrick, 2008 ▶); molecular graphics: *DIAMOND* (Brandenburg, 2006 ▶); software used to prepare material for publication: *SHELXL97*.

## Supplementary Material

Crystal structure: contains datablocks global, I. DOI: 10.1107/S1600536809043347/hb5159sup1.cif

Structure factors: contains datablocks I. DOI: 10.1107/S1600536809043347/hb5159Isup2.hkl

Additional supplementary materials:  crystallographic information; 3D view; checkCIF report

## Figures and Tables

**Table 1:** Hydrogen-bond geometry (Å, °)

*D*—H⋯*A*	*D*—H	H⋯*A*	*D*⋯*A*	*D*—H⋯*A*
N3—H3n⋯O3^i^	0.88	1.98	2.854 (3)	174
N4—H4n⋯N2^ii^	0.88	2.22	3.086 (3)	167

Symmetry codes: (i) 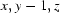; (ii) 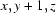.

**Fig. 1.**
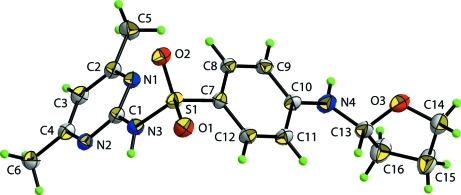
Molecular structure of (I) showing atom-labelling scheme and displacement ellipsoids at the 50% probability level.

**Fig. 2.**
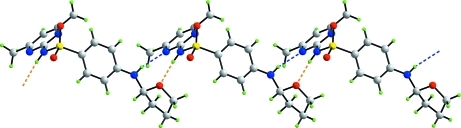
Supramolecular chain formation along the b axis in (I) mediated by N—H···N (orange dashed lines) and N—H···N (blue dashed lines) hydrogen bonding.

## supplementary crystallographic information

### Comment

The co-crystallization of active pharmaceutical ingredients is an active area of contemporary crystal engineering (Shan & Zaworotko, 2008). Sulfonamide drugs, *e.g.* sulfadimidine and sulfameter, attract significant interest in this regard, especially owing to their propensity to form polymorphs (Caira, 2007). They are also receiving renewed attention as selective inhibitors of carbonic anhydrase isoforms (*e.g.* Nishimori *et al.*, 2009). As a continuation of studies into the phenomenon of co-crystallization (Broker & Tiekink, 2008; Broker *et al.*, 2008), the co-crystallization of *N*'-(4,6-dimethyl-2-pyrimidinyl)sulfanilamide (sulfadimidine) and 1,4-C_6_H_4_I_2_ in THF was investigated. Colourless crystals of the title compound (I) were obtained unexpectedly; we are not aware of any precedence for this reaction. The insertion of nitrenes into the α C—H bond of cyclic ethers is known (Fructos *et al.*, 2006) and it is suggested that adventitious I_2_ in 1,4-C_6_H_4_I_2_ reacts with the aryl amine to give a nitrene stabilized by the *para*-sulfonamide group (Kemnitz *et al.*, 1998).

The molecule of (I), Fig. 1, is bent at the S atom, N3—S1—C7 = 107.85 (10)°, and adopts an overall `L'-conformation; the dihedral angle between the two six-membered rings is 76.93 (7)°. The five membered ring adopts an envelope configuration at the C16 atom. The crystal packing is dominated by N—H···O and N—H···N hydrogen bonding interactions, Table 1, that co-operate to form a supramolecular chain along the *b* axis, Fig. 2.

### Experimental

Colourless crystals of (I) were isolated from the attempted co-crystallization of *N*'-(4,6-dimethyl-2-pyrimidinyl)-sulfanilamide and 1,4-di-iodobenzene in THF.

### Refinement

Carbon-bound H-atoms were placed in calculated positions (C–H 0.95–1.00 Å) and were included in the refinement in the riding model approximation with *U*_iso_(H) set to 1.2–1.5*U*_eq_(C). The nitrogen-bound H-atoms were located in a difference Fourier map and were refined with a N–H 0.880±0.001 Å restraint, and with *U*_iso_(H) = 1.2*U*_eq_(N).

### Crystal data

**Table:**

C_16_H_20_N_4_O_3_S	*F*(000) = 736
*M**_r_* = 348.42	*D*_x_ = 1.422 Mg m^−^^3^
Monoclinic, *P*2_1_/*c*	Mo *K*α radiation, λ = 0.71073 Å
Hall symbol: -P 2ybc	Cell parameters from 6601 reflections
*a* = 10.291 (5) Å	θ = 2.5–40.2°
*b* = 9.592 (4) Å	µ = 0.22 mm^−^^1^
*c* = 17.196 (8) Å	*T* = 98 K
β = 106.445 (10)°	Block, colourless
*V* = 1628.0 (13) Å^3^	0.35 × 0.21 × 0.11 mm
*Z* = 4	

### Data collection

**Table:**

Saturn724 diffractometer	3749 independent reflections
Radiation source: sealed tube	3341 reflections with *I* > 2σ(*I*)
graphite	*R*_int_ = 0.046
Detector resolution: 28.5714 pixels mm^-1^	θ_max_ = 27.5°, θ_min_ = 2.1°
ω scans	*h* = −12→13
Absorption correction: multi-scan (*ABSCOR*; Higashi, 1995)	*k* = −12→11
*T*_min_ = 0.761, *T*_max_ = 1.000	*l* = −22→17
11164 measured reflections	

### Refinement

**Table:**

Refinement on *F*^2^	Primary atom site location: structure-invariant direct methods
Least-squares matrix: full	Secondary atom site location: difference Fourier map
*R*[*F*^2^ > 2σ(*F*^2^)] = 0.057	Hydrogen site location: inferred from neighbouring sites
*wR*(*F*^2^) = 0.137	H-atom parameters constrained
*S* = 1.10	*w* = 1/[σ^2^(*F*_o_^2^) + (0.0464*P*)^2^ + 1.4631*P*] where *P* = (*F*_o_^2^ + 2*F*_c_^2^)/3
3749 reflections	(Δ/σ)_max_ < 0.001
225 parameters	Δρ_max_ = 0.60 e Å^−^^3^
2 restraints	Δρ_min_ = −0.39 e Å^−^^3^

### Special details

**Table:**

Geometry. All s.u.'s (except the s.u. in the dihedral angle between two l.s. planes) are estimated using the full covariance matrix. The cell s.u.'s are taken into account individually in the estimation of s.u.'s in distances, angles and torsion angles; correlations between s.u.'s in cell parameters are only used when they are defined by crystal symmetry. An approximate (isotropic) treatment of cell s.u.'s is used for estimating s.u.'s involving l.s. planes.
Refinement. Refinement of *F*^2^ against ALL reflections. The weighted *R*-factor *wR* and goodness of fit *S* are based on *F*^2^, conventional *R*-factors *R* are based on *F*, with *F* set to zero for negative *F*^2^. The threshold expression of *F*^2^ > 2σ(*F*^2^) is used only for calculating *R*-factors(gt) *etc*. and is not relevant to the choice of reflections for refinement. *R*-factors based on *F*^2^ are statistically about twice as large as those based on *F*, and *R*- factors based on ALL data will be even larger.

### Fractional atomic coordinates and isotropic or equivalent isotropic displacement parameters (Å^2^)

**Table:**

	*x*	*y*	*z*	*U*_iso_*/*U*_eq_	
S1	0.31473 (6)	0.75837 (6)	0.11287 (3)	0.02795 (16)	
O1	0.23391 (18)	0.71472 (18)	0.03422 (9)	0.0357 (4)	
O2	0.45898 (17)	0.76422 (18)	0.12920 (9)	0.0339 (4)	
O3	0.03413 (17)	1.4900 (2)	0.10040 (10)	0.0406 (4)	
N1	0.40297 (19)	0.74868 (19)	0.29276 (10)	0.0271 (4)	
N2	0.27644 (18)	0.53811 (19)	0.29370 (10)	0.0265 (4)	
N3	0.2755 (2)	0.6434 (2)	0.17341 (10)	0.0283 (4)	
H3N	0.2048	0.5906	0.1512	0.034*	
N4	0.1102 (2)	1.3038 (2)	0.18799 (14)	0.0421 (5)	
H4N	0.1658	1.3594	0.2227	0.051*	
C1	0.3215 (2)	0.6443 (2)	0.25786 (12)	0.0258 (4)	
C2	0.4447 (2)	0.7458 (2)	0.37479 (12)	0.0283 (5)	
C3	0.4038 (2)	0.6408 (2)	0.41786 (12)	0.0298 (5)	
H3	0.4336	0.6395	0.4754	0.036*	
C4	0.3183 (2)	0.5378 (2)	0.37529 (12)	0.0283 (4)	
C5	0.5376 (3)	0.8614 (3)	0.41506 (14)	0.0378 (5)	
H5A	0.6280	0.8455	0.4085	0.057*	
H5B	0.5435	0.8637	0.4729	0.057*	
H5C	0.5021	0.9506	0.3901	0.057*	
C6	0.2706 (3)	0.4199 (3)	0.41651 (14)	0.0348 (5)	
H6A	0.1741	0.4032	0.3904	0.052*	
H6B	0.2840	0.4432	0.4737	0.052*	
H6C	0.3223	0.3357	0.4125	0.052*	
C7	0.2564 (2)	0.9212 (2)	0.13414 (12)	0.0279 (4)	
C8	0.3417 (2)	1.0138 (2)	0.18760 (13)	0.0291 (5)	
H8	0.4334	0.9891	0.2129	0.035*	
C9	0.2929 (2)	1.1411 (2)	0.20371 (13)	0.0307 (5)	
H9	0.3513	1.2038	0.2402	0.037*	
C10	0.1574 (2)	1.1793 (2)	0.16669 (13)	0.0318 (5)	
C11	0.0733 (2)	1.0854 (2)	0.11240 (14)	0.0344 (5)	
H11	−0.0181	1.1100	0.0862	0.041*	
C12	0.1224 (2)	0.9580 (2)	0.09687 (13)	0.0322 (5)	
H12	0.0645	0.8949	0.0605	0.039*	
C13	−0.0074 (3)	1.3735 (3)	0.14064 (16)	0.0384 (6)	
H13	−0.0624	1.3077	0.0991	0.046*	
C14	−0.0727 (3)	1.5920 (3)	0.0855 (2)	0.0582 (8)	
H14A	−0.1141	1.6035	0.0265	0.070*	
H14B	−0.0360	1.6832	0.1085	0.070*	
C15	−0.1763 (3)	1.5425 (4)	0.1246 (2)	0.0573 (8)	
H15A	−0.2534	1.4971	0.0848	0.069*	
H15B	−0.2105	1.6199	0.1513	0.069*	
C16	−0.0957 (3)	1.4381 (4)	0.1863 (2)	0.0618 (9)	
H16A	−0.0415	1.4851	0.2362	0.074*	
H16B	−0.1557	1.3681	0.2007	0.074*	

### Atomic displacement parameters (Å^2^)

**Table:**

	*U*^11^	*U*^22^	*U*^33^	*U*^12^	*U*^13^	*U*^23^
S1	0.0279 (3)	0.0329 (3)	0.0212 (2)	0.0002 (2)	0.0038 (2)	0.00097 (19)
O1	0.0393 (9)	0.0426 (10)	0.0218 (7)	−0.0014 (8)	0.0032 (7)	−0.0022 (6)
O2	0.0289 (8)	0.0431 (9)	0.0297 (8)	0.0015 (7)	0.0085 (7)	0.0009 (7)
O3	0.0274 (8)	0.0554 (11)	0.0384 (9)	0.0039 (8)	0.0083 (7)	0.0090 (8)
N1	0.0287 (9)	0.0260 (9)	0.0242 (8)	0.0000 (7)	0.0037 (7)	−0.0023 (7)
N2	0.0261 (9)	0.0278 (9)	0.0245 (8)	0.0005 (7)	0.0053 (7)	0.0001 (7)
N3	0.0307 (10)	0.0284 (9)	0.0221 (8)	−0.0028 (8)	0.0013 (7)	−0.0012 (7)
N4	0.0332 (11)	0.0337 (11)	0.0476 (12)	0.0010 (9)	−0.0079 (9)	−0.0087 (9)
C1	0.0250 (10)	0.0260 (10)	0.0246 (9)	0.0039 (8)	0.0042 (8)	−0.0011 (8)
C2	0.0297 (11)	0.0273 (10)	0.0251 (10)	0.0030 (9)	0.0031 (8)	−0.0039 (8)
C3	0.0329 (11)	0.0349 (12)	0.0198 (9)	0.0012 (9)	0.0044 (8)	−0.0017 (8)
C4	0.0277 (11)	0.0310 (11)	0.0265 (10)	0.0024 (9)	0.0080 (8)	0.0005 (8)
C5	0.0430 (14)	0.0333 (12)	0.0319 (11)	−0.0059 (11)	0.0020 (10)	−0.0070 (9)
C6	0.0336 (12)	0.0388 (13)	0.0311 (11)	−0.0018 (10)	0.0075 (10)	0.0042 (9)
C7	0.0262 (11)	0.0297 (11)	0.0253 (10)	−0.0007 (9)	0.0032 (8)	0.0046 (8)
C8	0.0238 (10)	0.0311 (11)	0.0286 (10)	−0.0031 (9)	0.0015 (8)	0.0048 (8)
C9	0.0264 (11)	0.0318 (11)	0.0290 (10)	−0.0064 (9)	−0.0001 (9)	0.0012 (8)
C10	0.0286 (11)	0.0305 (11)	0.0309 (11)	−0.0021 (9)	−0.0001 (9)	0.0012 (9)
C11	0.0268 (11)	0.0338 (12)	0.0355 (11)	−0.0005 (9)	−0.0025 (9)	0.0001 (9)
C12	0.0291 (11)	0.0341 (12)	0.0279 (10)	−0.0030 (9)	−0.0011 (9)	0.0000 (9)
C13	0.0300 (12)	0.0293 (12)	0.0472 (13)	−0.0007 (10)	−0.0031 (10)	−0.0046 (10)
C14	0.0399 (16)	0.0565 (19)	0.077 (2)	0.0120 (14)	0.0149 (15)	0.0303 (16)
C15	0.0522 (18)	0.063 (2)	0.0633 (18)	0.0242 (15)	0.0275 (15)	0.0178 (15)
C16	0.0483 (18)	0.073 (2)	0.074 (2)	0.0206 (16)	0.0344 (16)	0.0345 (18)

### Geometric parameters (Å, °)

**Table:**

S1—O2	1.4316 (18)	C6—H6A	0.9800
S1—O1	1.4351 (17)	C6—H6B	0.9800
S1—N3	1.644 (2)	C6—H6C	0.9800
S1—C7	1.748 (2)	C7—C12	1.392 (3)
O3—C14	1.439 (3)	C7—C8	1.396 (3)
O3—C13	1.442 (3)	C8—C9	1.379 (3)
N1—C1	1.334 (3)	C8—H8	0.9500
N1—C2	1.353 (3)	C9—C10	1.407 (3)
N2—C1	1.340 (3)	C9—H9	0.9500
N2—C4	1.346 (3)	C10—C11	1.405 (3)
N3—C1	1.394 (3)	C11—C12	1.377 (3)
N3—H3N	0.8800	C11—H11	0.9500
N4—C10	1.377 (3)	C12—H12	0.9500
N4—C13	1.420 (3)	C13—C16	1.494 (4)
N4—H4N	0.8800	C13—H13	1.0000
C2—C3	1.384 (3)	C14—C15	1.488 (4)
C2—C5	1.500 (3)	C14—H14A	0.9900
C3—C4	1.386 (3)	C14—H14B	0.9900
C3—H3	0.9500	C15—C16	1.523 (4)
C4—C6	1.490 (3)	C15—H15A	0.9900
C5—H5A	0.9800	C15—H15B	0.9900
C5—H5B	0.9800	C16—H16A	0.9900
C5—H5C	0.9800	C16—H16B	0.9900
			
O2—S1—O1	119.23 (10)	C8—C7—S1	121.15 (17)
O2—S1—N3	109.23 (10)	C9—C8—C7	120.0 (2)
O1—S1—N3	102.72 (10)	C9—C8—H8	120.0
O2—S1—C7	108.79 (11)	C7—C8—H8	120.0
O1—S1—C7	108.43 (10)	C8—C9—C10	120.7 (2)
N3—S1—C7	107.85 (10)	C8—C9—H9	119.6
C14—O3—C13	107.26 (19)	C10—C9—H9	119.6
C1—N1—C2	115.27 (19)	N4—C10—C11	122.3 (2)
C1—N2—C4	115.51 (18)	N4—C10—C9	118.9 (2)
C1—N3—S1	125.67 (16)	C11—C10—C9	118.6 (2)
C1—N3—H3N	116.9	C12—C11—C10	120.4 (2)
S1—N3—H3N	115.6	C12—C11—H11	119.8
C10—N4—C13	124.3 (2)	C10—C11—H11	119.8
C10—N4—H4N	119.7	C11—C12—C7	120.4 (2)
C13—N4—H4N	112.9	C11—C12—H12	119.8
N1—C1—N2	128.24 (19)	C7—C12—H12	119.8
N1—C1—N3	117.29 (19)	N4—C13—O3	108.7 (2)
N2—C1—N3	114.47 (18)	N4—C13—C16	116.1 (2)
N1—C2—C3	121.2 (2)	O3—C13—C16	103.8 (2)
N1—C2—C5	116.0 (2)	N4—C13—H13	109.3
C3—C2—C5	122.79 (19)	O3—C13—H13	109.3
C2—C3—C4	118.66 (19)	C16—C13—H13	109.3
C2—C3—H3	120.7	O3—C14—C15	108.2 (2)
C4—C3—H3	120.7	O3—C14—H14A	110.1
N2—C4—C3	121.1 (2)	C15—C14—H14A	110.1
N2—C4—C6	116.5 (2)	O3—C14—H14B	110.1
C3—C4—C6	122.36 (19)	C15—C14—H14B	110.1
C2—C5—H5A	109.5	H14A—C14—H14B	108.4
C2—C5—H5B	109.5	C14—C15—C16	101.9 (2)
H5A—C5—H5B	109.5	C14—C15—H15A	111.4
C2—C5—H5C	109.5	C16—C15—H15A	111.4
H5A—C5—H5C	109.5	C14—C15—H15B	111.4
H5B—C5—H5C	109.5	C16—C15—H15B	111.4
C4—C6—H6A	109.5	H15A—C15—H15B	109.3
C4—C6—H6B	109.5	C13—C16—C15	101.4 (2)
H6A—C6—H6B	109.5	C13—C16—H16A	111.5
C4—C6—H6C	109.5	C15—C16—H16A	111.5
H6A—C6—H6C	109.5	C13—C16—H16B	111.5
H6B—C6—H6C	109.5	C15—C16—H16B	111.5
C12—C7—C8	119.9 (2)	H16A—C16—H16B	109.3
C12—C7—S1	118.99 (17)		
			
O2—S1—N3—C1	56.4 (2)	N3—S1—C7—C8	95.14 (19)
O1—S1—N3—C1	−176.07 (18)	C12—C7—C8—C9	0.4 (3)
C7—S1—N3—C1	−61.7 (2)	S1—C7—C8—C9	−179.57 (16)
C2—N1—C1—N2	0.0 (3)	C7—C8—C9—C10	−0.1 (3)
C2—N1—C1—N3	179.72 (19)	C13—N4—C10—C11	−22.4 (4)
C4—N2—C1—N1	0.5 (3)	C13—N4—C10—C9	161.0 (2)
C4—N2—C1—N3	−179.25 (19)	C8—C9—C10—N4	176.2 (2)
S1—N3—C1—N1	1.0 (3)	C8—C9—C10—C11	−0.6 (3)
S1—N3—C1—N2	−179.18 (16)	N4—C10—C11—C12	−175.7 (2)
C1—N1—C2—C3	−0.2 (3)	C9—C10—C11—C12	0.9 (4)
C1—N1—C2—C5	179.3 (2)	C10—C11—C12—C7	−0.6 (4)
N1—C2—C3—C4	−0.1 (3)	C8—C7—C12—C11	0.0 (3)
C5—C2—C3—C4	−179.6 (2)	S1—C7—C12—C11	179.91 (18)
C1—N2—C4—C3	−0.8 (3)	C10—N4—C13—O3	−104.0 (3)
C1—N2—C4—C6	−179.43 (19)	C10—N4—C13—C16	139.5 (3)
C2—C3—C4—N2	0.6 (3)	C14—O3—C13—N4	−153.7 (2)
C2—C3—C4—C6	179.2 (2)	C14—O3—C13—C16	−29.5 (3)
O2—S1—C7—C12	156.85 (17)	C13—O3—C14—C15	5.4 (3)
O1—S1—C7—C12	25.8 (2)	O3—C14—C15—C16	20.2 (4)
N3—S1—C7—C12	−84.79 (19)	N4—C13—C16—C15	160.5 (3)
O2—S1—C7—C8	−23.2 (2)	O3—C13—C16—C15	41.3 (3)
O1—S1—C7—C8	−154.31 (18)	C14—C15—C16—C13	−37.0 (4)

### Hydrogen-bond geometry (Å, °)

**Table:**

*D*—H···*A*	*D*—H	H···*A*	*D*···*A*	*D*—H···*A*
N3—H3n···O3^i^	0.88	1.98	2.854 (3)	174
N4—H4n···N2^ii^	0.88	2.22	3.086 (3)	167

Symmetry codes: (i) *x*, *y*−1, *z*; (ii) *x*, *y*+1, *z*.
